# Identification of candidate biomarkers and pathways associated with psoriasis using bioinformatics analysis

**DOI:** 10.1186/s41065-020-00141-1

**Published:** 2020-07-15

**Authors:** Yongqi Luo, Yangyang Luo, Jing Chang, Zhenghui Xiao, Bin Zhou

**Affiliations:** 1 Department of Dermatology, Hunan Children’s Hospital, No. 86 Ziyuan Road, Yuhua District, Changsha, 410007 Hunan China; 2 Emergency Center, Hunan Children’s Hospital, Changsha, 410007 Hunan China

**Keywords:** Psoriasis, Pathogenesis, Differentially expressed genes, Risk subpathway analysis, Protein-protein network analysis

## Abstract

**Background:**

The aim of this study was to identify the candidate biomarkers and pathways associated with psoriasis. GSE13355 and GSE14905 were extracted from the Gene Expression Omnibus (GEO) database. Then the differentially expressed genes (DEGs) with |logFC| > 2 and adjusted *P* < 0.05 were chosen. In addition, the Gene ontology (GO) and Kyoto Encyclopedia of Genes and Genomes (KEGG) pathway enrichment analyses for DEGs were performed. Then, the GO terms with *P <* 0.05 and overlap coefficient greater than 0.5 were integrated by EnrichmentMap. Additionally, risk subpathways analysis for DEGs was also conducted by using the iSubpathwayMiner package to obtain more psoriasis-related DEGs and pathways. Finally, protein-protein interaction (PPI) network analysis was performed to identify the hub genes, and the DGIdb database was utilized to search for the candidate drugs for psoriasis.

**Results:**

A total of 127 DEGs which were mostly associated with keratinization, keratinocyte differentiation, and epidermal cell differentiation biological processes were identified. Based on these GO terms, 3 modules (human skin, epidermis and cuticle differentiation, and enzyme activity) were constructed. Moreover, 9 risk subpathways such as steroid hormone biosynthesis, folate biosynthesis, and pyrimidine metabolism were screened. Finally, PPI network analysis demonstrated that *CXCL10* was the hub gene with the highest degree, and *CXCR2*, *CXCL10*, *IVL*, *OASL*, and *ISG15* were the potential gene targets of the drugs for treating psoriasis.

**Conclusion:**

Psoriasis may be mostly caused by keratinization, keratinocyte differentiation, and epidermal cell differentiation; the pathogeneses were more related with pathways such as steroid hormone biosynthesis, folate biosynthesis, and pyrimidine metabolism. Besides, some psoriasis-related genes such as *SPRR* genes, *HSD11B1*, *GGH*, *CXCR2*, *IVL*, *OASL*, *ISG15*, and *CXCL10* may be important targets in psoriatic therapy.

## Background

Psoriasis is a common, chronic, relapsing, and immune-mediated skin disease, prevalent in 2–4% of the total population [1]. Psoriasis is not purely a skin disorder and it may also affect many other organs and cause other diseases [2–4]. The cause of psoriasis in nearly one-third of the patients is hereditary, though it is often caused or influenced by environmental factors [5]. Oxidative stress, stress, and systemic corticosteroid withdrawal are thought to be the contributing factors to psoriasis [6]. Therefore, exploring the possible mechanisms of psoriasis is of great importance.

Previous studies have reported that psoriasis is generally caused by aberrant keratinocyte proliferation and differentiation, epidermal hyperplasia, angiogenesis, dendritic cells and neutrophils, infiltration of T lymphocytes, and elements of innate immunity [7]. Also, many studies focused on the identification of psoriasis susceptibility loci which were correlated with the pathogenesis. For instance, Zhang et al. investigated psoriasis susceptibility loci in psoriatic arthritis and a generalized pustular psoriasis cohort, and found 3 significant loci, and the most significant single nucleotide polymorphism (SNP) was rs7709212 in 5q33.3 [8]. Sheng et al. confirmed 4 known psoriasis susceptibility loci (IL12B, IFIH1, ERAP1 and RNF114; 2.30 × 10^− 20^ ≤ *P* ≤ 2.41 × 10^− 7^) and identified 3 new susceptibility loci: 4q24 (NFKB1) at rs1020760 (*P* = 2.19 × 10^− 8^), 12p13.3 (CD27-LAG3) at rs758739 (*P* = 4.08 × 10^− 8^) and 17q12 (IKZF3) at rs10852936 (*P* = 1.96 × 10^− 8^) [9]. Nevertheless, the molecular mechanisms of psoriasis remain to be fully understood.

**Fig. 1:**
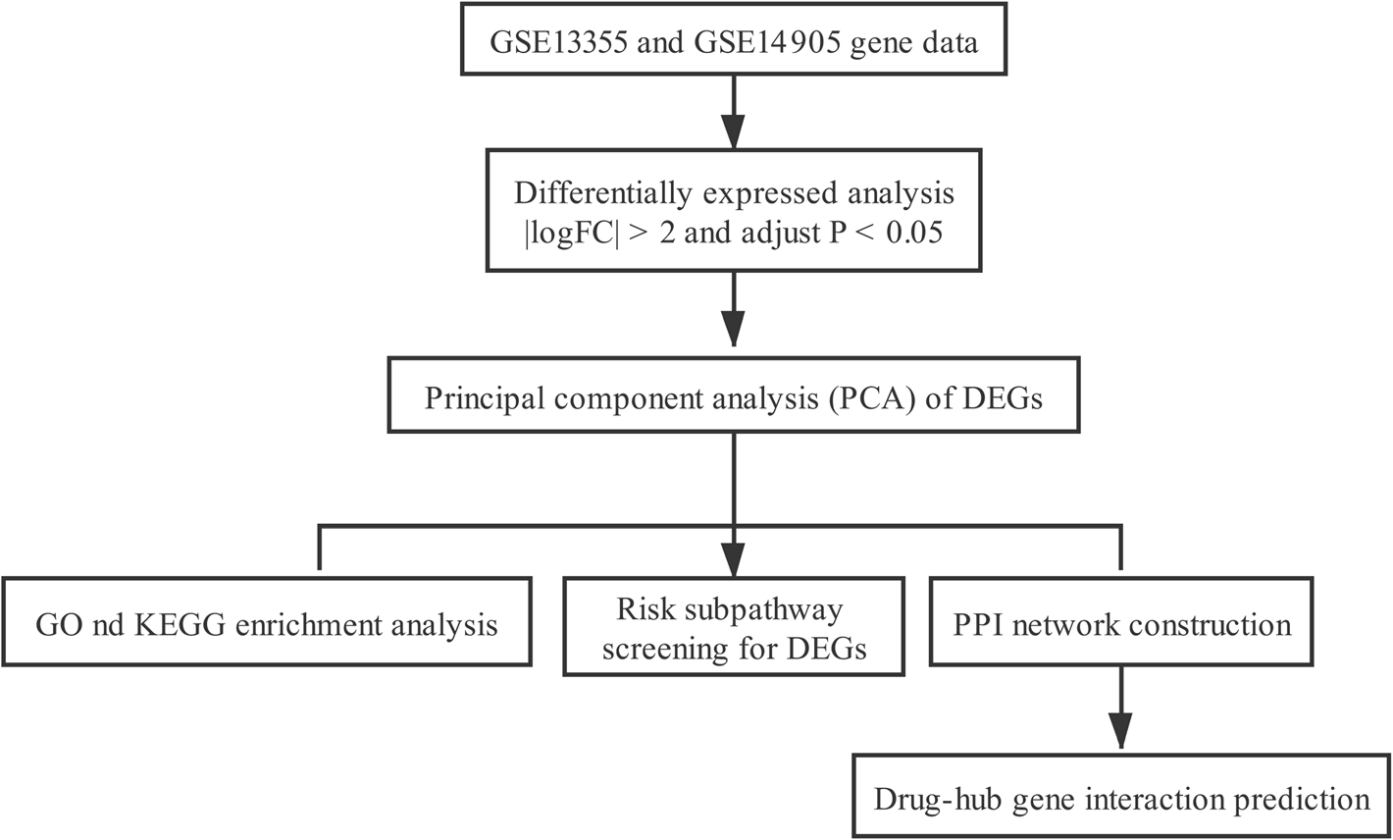
Flow chart of this study

Nair et al. [10] identified psoriasis susceptibility loci and Swindell et al. [11] used genome-wide transcriptional analysis to compare gene expression patterns in human psoriatic lesions with those in 5 mouse models of psoriasis. However, they had not performed in-depth bioinformatics analyses to explore the rehabilitation mechanisms of psoriasis. Using the gene sets (GSE13355 and GSE14905) deposited by Nair et al. and Swindell et al., the gene expression profiling data related to psoriasis were used to generate the differentially expressed genes (DEGs) involved in psoriasis. Moreover, the gene ontology (GO) term enrichment analysis, Kyoto Encyclopedia of Genes and Genomes (KEGG) pathway analysis, risk subpathway analysis, and construction of protein-protein interaction (PPI) network were all performed, and the DGIdb database was utilized to search for the candidate drugs for psoriasis. Finally, the DEGs and pathways mostly correlated with psoriasis were analyzed to investigate the pathogenesis. Therefore, our study provides a deep understanding of susceptibility genes in psoriasis and may provide appropriate drug targets for psoriatic therapies. A flow chart depicting the schematic of this study has been shown in Fig. 1.

## Results

### Differentially expressed genes between normal and lesional groups

**Fig. 2:**
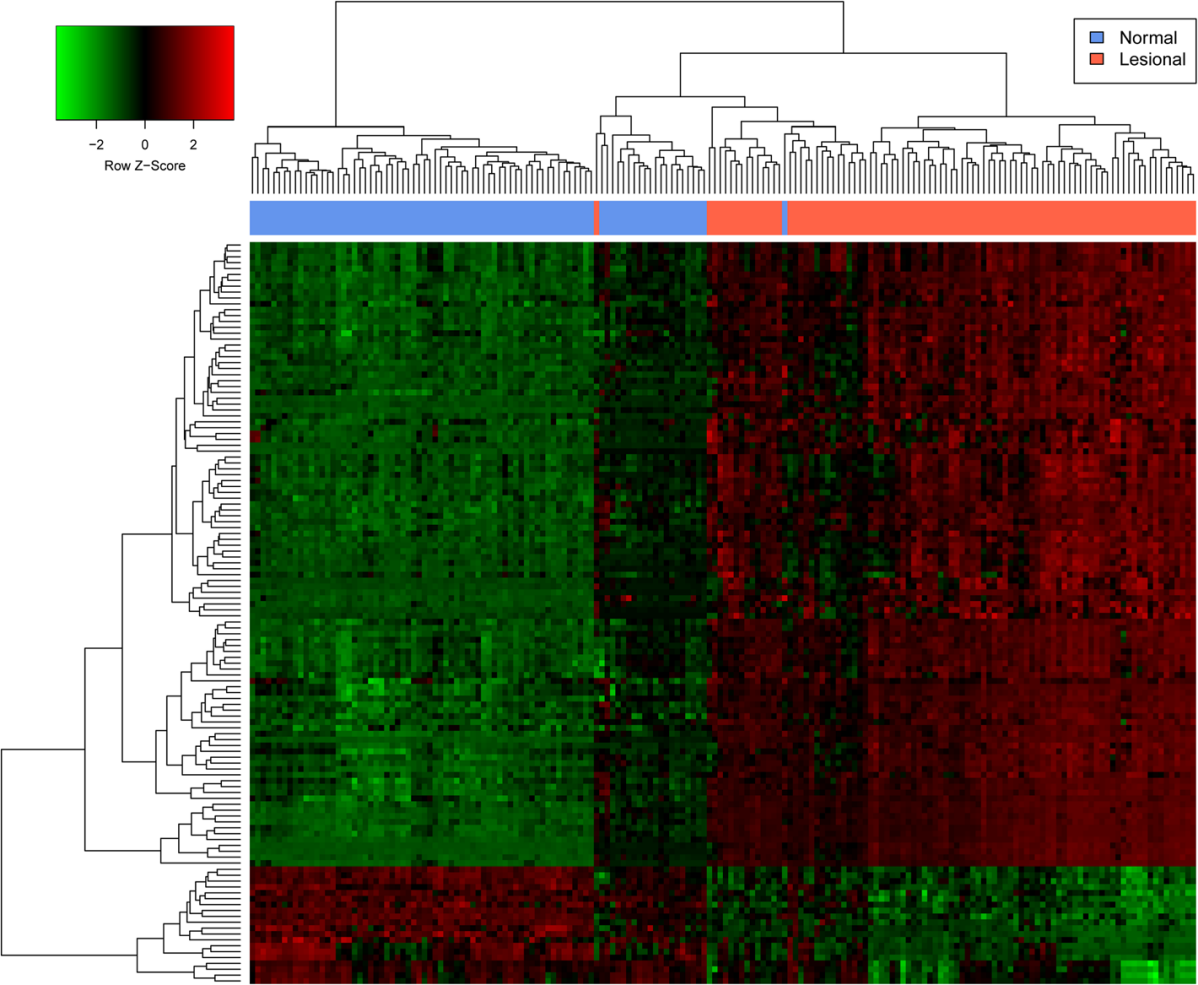
Heatmap of DEGs. The red and green colors represent up-regulation and down-regulation, respectively

A total of 127 DEGs were screened between normal and lesional groups with |logFC| > 2 and adjusted *P* < 0.05, including 97 (76%) up-regulated DEGs and 30 (24%) down-regulated DEGs (Supplementary Table 1). The heatmap of the DEGs has been shown in Fig. 2, and the result shows that the two groups could be significantly separated, indicating that the results of the difference analysis were reliable.

### Principal component analysis of differentially expressed genes

**Fig. 3:**
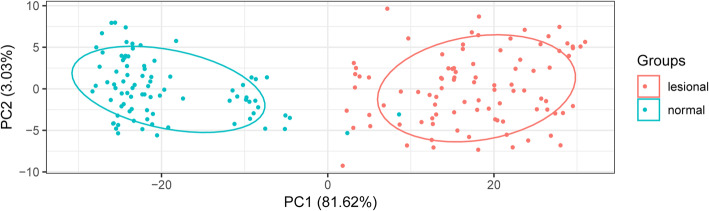
PCA between the psoriasis and normal control groups

As presented in Fig. 3, lesional and normal control samples were completely separated by the selected DEGs, meaning that the expression patterns of screened DEGs were specific and could be used to completely distinguish between lesional and normal control samples.

### Functional enrichment analysis of differentially expressed genes

**Fig. 4:**
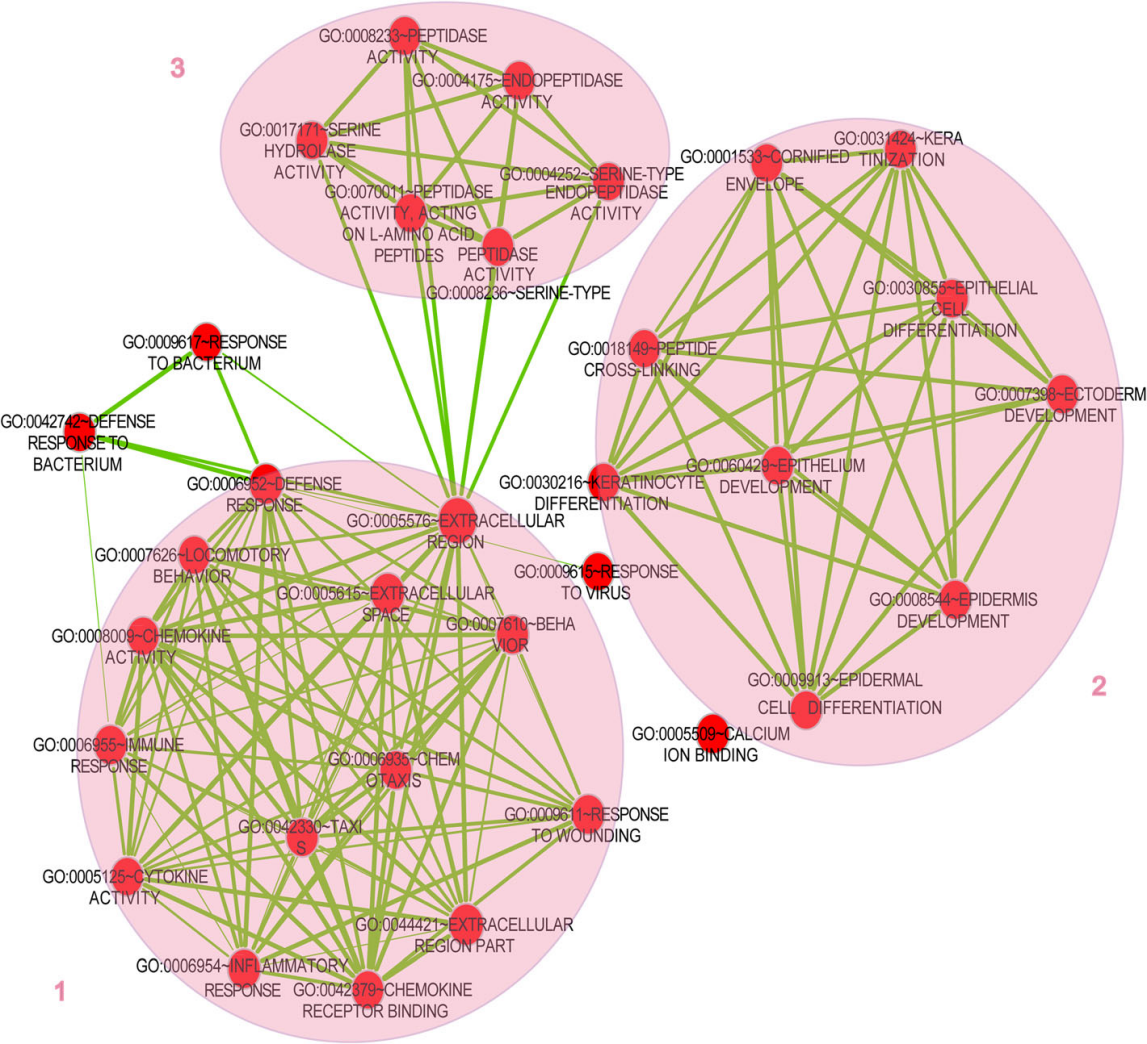
Integration of GO terms for differentially expressed genes (DEGs). Red nodes represent GO terms. Green lines indicate that overlapping DEGs exist between two GO terms. Three circles represent three modules: module 1 mostly associates with skin, module 2 mostly associates with epidermis and cuticle differentiation, and module 3 mostly connects with enzyme activity

Functional enrichment analyses of the GO terms and KEGG pathway were performed for both up-regulated and down-regulated DEGs. All the DEGs were mainly enriched in 48 GO terms, containing 30 ontology terms in biological process, and the top 10 GO terms in biological process have been shown in Table 1. Biological processes of DEGs were mostly associated with keratinization (GO: 0031424), keratinocyte differentiation (GO: 0030216), and epidermal cell differentiation (GO: 0009913). By integrating these GO terms using EnrichmentMap, 3 modules were obtained, which mostly associated with human skin (module 1), epidermis and cuticle differentiation (module 2), and enzyme activity (module 3) (Fig. 4). In the KEGG pathway analysis, the DEGs were enriched in 4 pathways and most significantly related to the chemokine signaling pathway (10 genes, hsa04062), cytokine-cytokine receptor interaction (9 genes, hsa04060), toll-like receptor signaling pathway (6 genes, hsa04620), and RIG-I-like receptor signaling pathway (4 genes, hsa04622) (Table 2). The enrichment analysis showed that small proline-rich protein (*SPRR*, including *SPRR2C*, *SPRR1A* and *SPRR2B*) genes were involved in all the biological processes related to epidermis and cuticle differentiation (Table 1). Moreover, CXC chemokine ligand (*CXCL*, including *CXCL1*, *CXCL9* and *CXCL10*) genes were mostly involved in human skin-related GO terms and 4 KEGG pathways, and the C-X-C motif chemokine receptor 2 (*CXCR2*) was involved in the chemokine signaling pathway and cytokine-cytokine receptor interaction (Table 1 and Table 2).

**Table 1:** Top 10 GO terms in biological process for differentially expressed genes (DEGs) in gene expression profile of psoriatic patients (*P* < 0.05)

ID	Name	Pvalue	DEGs
GO:0031424	Keratinization	6.15E-10	*SPRR2C*, *SPRR1A*, *SPRR1B*, *LCE3D*, *TGM1*, *CNFN*, *SPRR3*, *TGM3*, *SPRR2G*, *IVL*
GO:0030216	Keratinocyte differentiation	9.07E-10	*SPRR2C*, *S100A7*, *SPRR1A*, *SPRR1B*, *LCE3D*, *TGM1*, *CNFN*, *SPRR3*, *TGM3*, *SPRR2G*, *IVL*
GO:0009913	Epidermal cell differentiation	2.01E-09	*SPRR2C*, *S100A7*, *SPRR1A*, *SPRR1B*, *LCE3D*, *TGM1*, *CNFN*, *SPRR3*, *TGM3*, *SPRR2G*, *IVL*
GO:0007398	Ectoderm development	1.76E-08	*KRT6A*, *S100A7*, *LCE3D*, *SPRR2G*, *SPRR2C*, *KRT16*, *SPRR1A*, *SPRR1B*, *CNFN*, *TGM1*, *SPRR3*, *TGM3*, *ALOX12B*, *IVL*
GO:0030855	Epithelial cell differentiation	4.85E-08	*RHCG*, *SPRR2C*, *S100A7*, *SPRR1A*, *SPRR1B*, *LCE3D*, *TGM1*, *CNFN*, *SPRR3*, *TGM3*, *SPRR2G*, *IVL*
GO:0006952	Defense response	5.96E-08	*CXCL1*, *DCD*, *KYNU*, *IL8*, *S100A8*, *S100A7*, *S100A9*, *CXCL9*, *RSAD2*, *CXCR2*, *CCL18*, *S100A12*, *CXCL10*, *WFDC12*, *FOS*, *CCL20*, *CXCL13*, *IRF7*, *LTF*, *MX1*
GO:0008544	Epidermis development	7.87E-08	*S100A7*, *LCE3D*, *SPRR2G*, *SPRR2C*, *SPRR1A*, *KRT16*, *SPRR1B*, *CNFN*, *TGM1*, *SPRR3*, *TGM3*, *ALOX12B*, *IVL*
GO:0060429	Epithelium development	6.53E-07	*S100A7*, *LCE3D*, *SPRR2G*, *GREM1*, *SPRR2C*, *RHCG*, *SPRR1A*, *SPRR1B*, *CNFN*, *TGM1*, *SPRR3*, *TGM3*, *IVL*
GO:0006935	Chemotaxis	2.12E-06	*CXCL1*, *IL8*, *CCL20*, *CXCL13*, *S100A9*, *CXCL9*, *CXCR2*, *CCL27*, *CCL18*, *CXCL10*
GO:0042330	Taxis	2.12E-06	*CXCL1*, *IL8*, *CCL20*, *CXCL13*, *S100A9*, *CXCL9*, *CXCR2*, *CCL27*, *CCL18*, *CXCL10*

**Table 2:** KEGG pathway enrichment analysis of differentially expressed genes (DEGs) in gene expression profile of psoriatic patients (*P* < 0.05)

ID	Name	Pvalue	DEGs
Hsa04062	Chemokine signaling pathway	5.17E-06	*CXCL1*, *IL8*, *CCL20*, *CXCL13*, *CXCL9*, *CXCR2*, *STAT1*, *CCL27*, *CCL18*, *CXCL10*
Hsa04060	Cytokine-cytokine receptor interaction	4.67E-04	*CXCL1*, *IL8*, *CCL20*, *CXCL13*, *CXCL9*, *CXCR2*, *CCL27*, *CCL18*, *CXCL10*
Hsa04620	Toll-like receptor signaling pathway	7.37E-04	*FOS*, *IL8*, *IRF7*, *CXCL9*, *STAT1*, *CXCL10*
Hsa04622	RIG-I-like receptor signaling pathway	0.014435	*ISG15*, *IL8*, *IRF7*, *CXCL10*

### Risk subpathway enrichment analysis of differentially expressed genes

A total of 9 risk subpathways were identified by using the isubpathwayMiner package (Table 3). The three most significant subpathways were steroid hormone biosynthesis (path:00140_13) (*P* = 0.01561), folate biosynthesis (path:00790_4) (*P* = 0.01561), and pyrimidine metabolism (path:00240_21) (*P* = 0.019471). The enzyme 11beta-hydroxysteroid dehydrogenase type 1 (*HSD11B1*) is involved in steroid hormone biosynthesis, and the enzyme gamma-glutamyl hydrolase (*GGH*) is involved in folate biosynthesis.

**Table 3:** Risk subpathway enrichment analysis of differentially expressed genes (DEGs) in psoriatic patients (*P* < 0.05)

ID	Name	Pvalue	DEGs
Path:00140_13	Steroid hormone biosynthesis	0.01561	*HSD11B1*
Path:00790_4	Folate biosynthesis	0.01561	*GGH*
Path:00240_21	Pyrimidine metabolism	0.019471	*CMPK2*, *RRM2*
Path:00240_16	Pyrimidine metabolism	0.030339	*CMPK2*, *RRM2*
Path:00531_3	Glycosaminoglycan degradation	0.041091	*HYAL4*
Path:00240_12	Pyrimidine metabolism	0.044307	*CMPK2*, *RRM2*
Path:00140_18	Steroid hormone biosynthesis	0.046108	*HSD11B1*
Path:00380_10	Tryptophan metabolism	0.046108	*KYNU*
Path:00480_6	Glutathione metabolism	0.046108	*RRM2*

### Construction of protein-protein interaction network based on differentially expressed genes

**Fig. 5:**
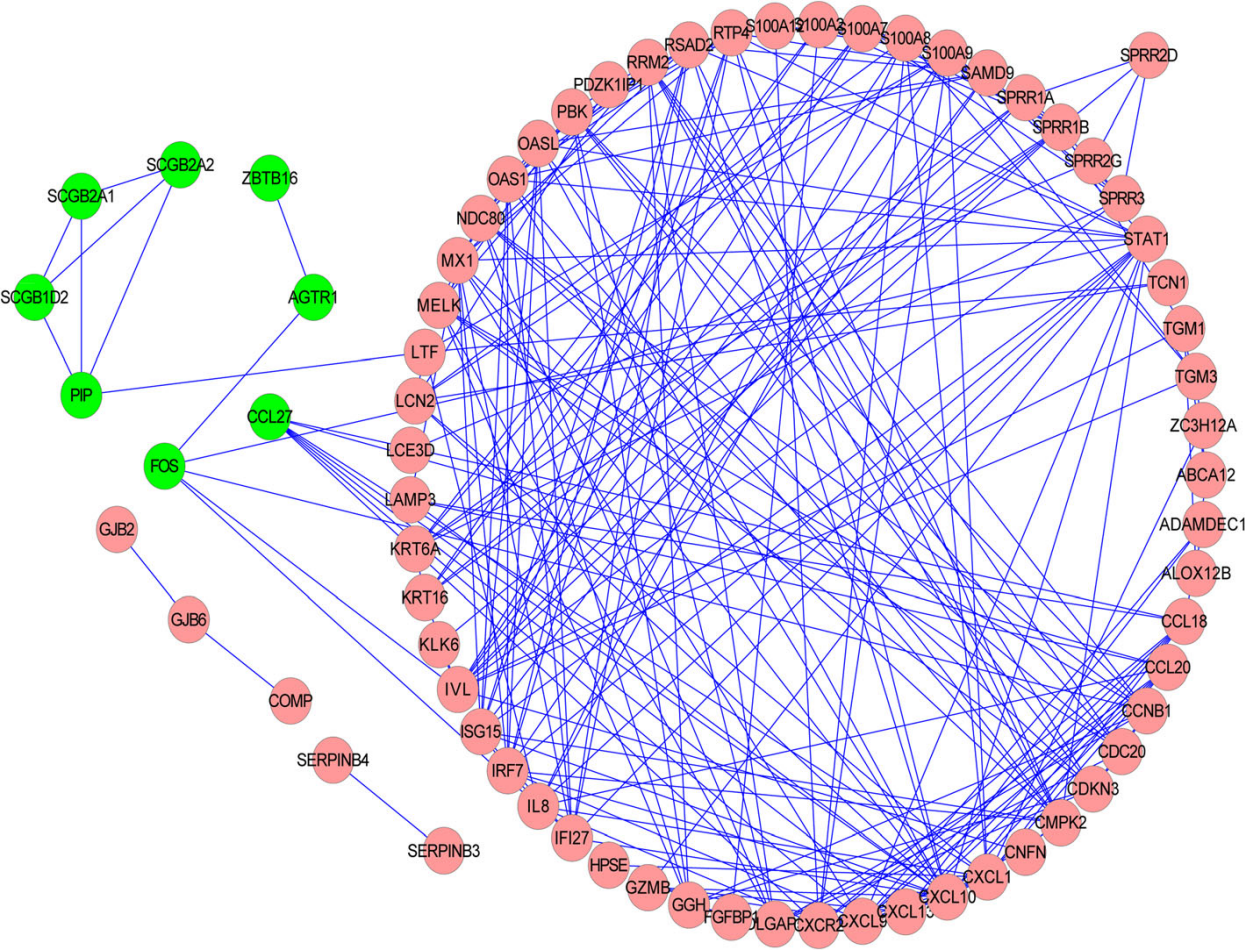
The protein-protein interaction (PPI) network constructed on the interactions of protein molecules in psoriatic patients. Pink nodes represent the up-regulated DEGs; Green nodes represent the down-regulated DEGs; the edges represent the interactions between proteins

To explore the interactions among psoriasis-related DEGs, the PPI network containing 71 nodes and 225 edges was constructed (Fig. 5). Of all the nodes, only 8 nodes were down-regulated DEGs, while the other 63 nodes were up-regulated DEGs. In this PPI network, the top 16 proteins with higher degrees were chosen, among which CXCL10 had the highest degree (Table 4).

**Table 4:** Top 16 proteins of the higher degrees in protein-protein interaction (PPI) network of differentially expressed genes (DEGs)

Proteins	Degree
CXCL10	20
STAT1	15
CXCL1	13
CXCL9	12
IRF7	12
IVL	12
MX1	12
S100A9	12
CXCR2	11
ISG15	11
OAS1	11
OASL	11
RSAD2	11
RTP4	11
SPRR1B	11
CMPK2	10

### Drug-hub gene interaction

**Fig. 6:**
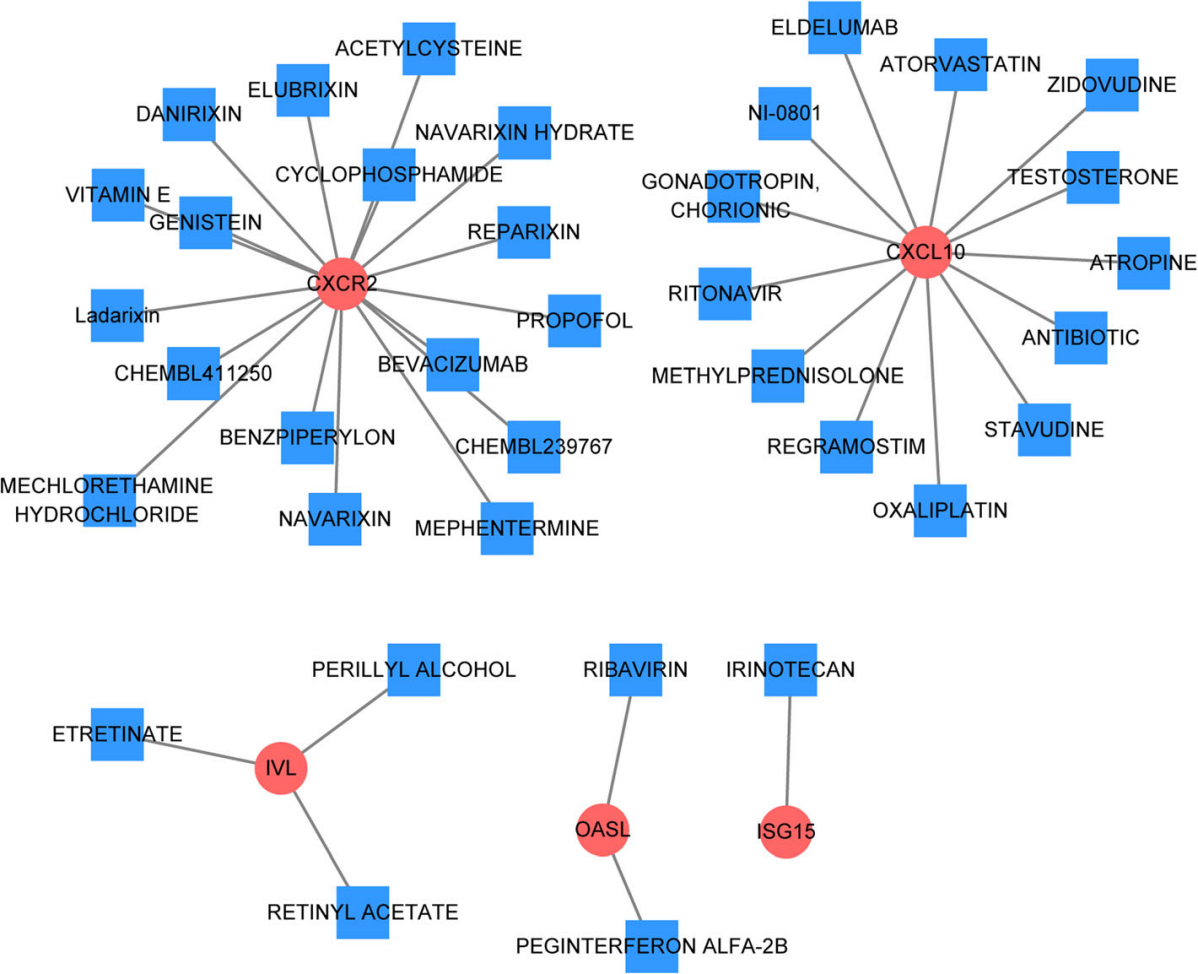
Drug-hub gene interaction. Red nodes represent the up-regulated DEGs; Blue squares represent the drugs

Using the 16 hub genes to explore the drug-gene interactions, 36 drugs for possibly treating psoriasis were compiled and selected (Fig. 6 and Table 5). Promising targets of these drugs included *CXCR2*, *CXCL10*, involucrin (*IVL*), 2'-5'-oligoadenylate synthetase like (*OASL*), and ISG15 ubiquitin-like modifier (*ISG15*).

**Table 5:** Candidate drugs targeting hub genes

gene	drug	interaction_types	sources	pmids
CXCR2	PROPOFOL	agonist	GuideToPharmacologyInteractions	–
CXCR2	BENZPIPERYLON	agonist	GuideToPharmacologyInteractions	–
CXCR2	CHEMBL411250	agonist	GuideToPharmacologyInteractions	–
CXCR2	MEPHENTERMINE	agonist	GuideToPharmacologyInteractions	–
CXCR2	REPARIXIN	allosteric modulator|modulator|antagonist	GuideToPharmacologyInteractions|ChemblInteractions|TTD	–
CXCR2	DANIRIXIN	antagonist	GuideToPharmacologyInteractions|ChemblInteractions	–
CXCR2	NAVARIXIN	antagonist	GuideToPharmacologyInteractions|ChemblInteractions|TTD	–
CXCR2	CHEMBL239767	antagonist	GuideToPharmacologyInteractions	–
CXCR2	NAVARIXIN HYDRATE	antagonist	ChemblInteractions	–
CXCR2	ELUBRIXIN	antagonist	ChemblInteractions|TTD	–
CXCR2	Ladarixin	modulator	ChemblInteractions	–
CXCR2	BEVACIZUMAB	–	PharmGKB	–
CXCR2	CYCLOPHOSPHAMIDE	–	PharmGKB	–
CXCR2	ACETYLCYSTEINE	–	NCI	10,716,998
CXCR2	MECHLORETHAMINE HYDROCHLORIDE	–	NCI	12,753,603
CXCR2	VITAMIN E	–	NCI	16,679,868
CXCR2	GENISTEIN	–	NCI	9,712,063
ISG15	IRINOTECAN	inhibitor	MyCancerGenomeClinicalTrial	–
OASL	PEGINTERFERON ALFA-2B	–	PharmGKB	–
OASL	RIBAVIRIN	–	PharmGKB	–
IVL	ETRETINATE	–	NCI	12,445,200|8,381,448
IVL	RETINYL ACETATE	–	NCI	6,189,593
IVL	PERILLYL ALCOHOL	–	NCI	10,769,631
CXCL10	NI-0801	inhibitor	ChemblInteractions	–
CXCL10	METHYLPREDNISOLONE	–	NCI	17,220,550
CXCL10	TESTOSTERONE	–	NCI	9,681,518
CXCL10	REGRAMOSTIM	–	NCI	11,591,765
CXCL10	OXALIPLATIN	–	NCI	16,101,140
CXCL10	ANTIBIOTIC	–	NCI	10,634,213
CXCL10	ZIDOVUDINE	–	NCI	11,141,242
CXCL10	ATROPINE	–	NCI	15,315,164
CXCL10	ELDELUMAB	–	TdgClinicalTrial	–
CXCL10	STAVUDINE	–	NCI	11,141,242
CXCL10	GONADOTROPIN, CHORIONIC	–	NCI	15,590,984
CXCL10	RITONAVIR	–	NCI	11,141,242
CXCL10	ATORVASTATIN	–	NCI	10,559,511

## Discussion

Psoriasis is a common skin disease, and it is an organ-specific autoimmune disease which is a common immune-mediated skin disease in adults [12]. Numerous studies have reported that psoriasis is generally caused by aberrant keratinocyte proliferation and epidermal hyperplasia [7, 13]. In this study, GO terms in biological processes (e.g. keratinization, keratinocyte differentiation, and epidermal cell differentiation) for DEGs were mostly associated with epidermis and cuticle differentiation, and the *SPRR* gene family were the most correlated DEGs. *SPRR* genes, consisting of two genes for *SPRR1* (*SPRR1A* and *SPRR1B*), seven genes for *SPRR2*, and a single gene for *SPRR3*, encode a novel class of small proline-rich proteins that could be strongly induced during the differentiation of epidermal keratinocytes [14]. Kainu et al. found that three candidate gene clusters, the *S100*, *SPRR,* and *PGLYRP* genes, contained functionally interesting psoriasis candidate genes [15], which were consistent with our results. Besides, the KEGG pathway enrichment analysis showed that *CXCR2* was involved in the chemokine signaling pathway and cytokine-cytokine receptor interaction. It has been shown that the expression of epidermal *CXCR2* is increased in psoriasis, suggesting that activation of keratinocytes mediated by *CXCR2* contributes to the characteristic epidermal changes observed in psoriasis [16]. The results of this study showed that *CXCR2* was the potential gene target of the drugs for treating psoriasis. Moreover, proinflammatory cytokines are found to be associated with the progression of cerebral white matter injury (WMI) in preterm infants, and cytokine-receptor interaction may be critical in determining the effects of inflammation in the development of the disease, which further suggested that *CXCR2* might have been related to the development of psoriasis via the chemokine signaling pathway and cytokine-cytokine receptor interaction.

Through risk subpathway enrichment analysis, subpathways such as steroid hormone biosynthesis, folate biosynthesis, and pyrimidine metabolism were identified, and *HSD11B1* wass involved in steroid hormone biosynthesis, and *GGH* was involved in folate biosynthesis. *HSD11B1* is the main regulator of tissue-specific effects of excessive circulating glucocorticoids, which may be related to inflammation [17, 18]. Thus, *HSD11B1* was connected with psoriasis which has been always recognized as a chronic inflammatory disease of the skin. Additionally, Sevilla et al. showed that *HSD11B1* was associated with osteoporosis, and *HSD11B1*/*HSD11B2* were responsible for cortisol to cortisone interconversion [19]. Therefore, *HSD11B1* might be recognized as the probable gene associated with psoriasis. Furthermore, Warren et al. reported that *GGH* was an important target gene of psoriasis which was involved in folate biosynthesis from our study [20]. Pyrimidine metabolism was another important subpathway identified in the current study. Zhou et al. constructed a ceRNA network to determine the regulatory roles of lncRNAs in psoriasis, and found that the upregulated mRNAs were mainly enriched in the pyrimidine metabolism, cell cycle, and cytokine-cytokine receptor interaction signaling pathways [21]. In summary, DEGs involved in risk subpathways were more likely related to psoriasis and might be important targets in psoriatic therapy. Also, all the subpathways identified by risk subpathway analysis were considered as the most relevant pathways with psoriasis.

The PPI network analysis showed that protein with the highest degree was encoded by *CXCL10*, a member of the CXC chemokine family of cytokines [22]. The results of this study showed that *CXCL10* was the potential gene target of the drugs for treating psoriasis. *CXCL10* has been reported to be expressed in many Th1-type human inflammatory diseases, including skin diseases (e.g. psoriasis) [23]. A recent study suggested that the decrease in serum *CXCL10* level over time was related to the new onset of psoriatic arthritis in patients with psoriasis [24], and our study revealed a same result that *CXCL10* was up-regulated in psoriasis. In the present study, the CXC chemokine family of cytokines (containing *CXCL1*, *CXCL9,* and *CXCL10*) was mostly enriched in human skin-related GO terms and KEGG pathways such as the chemokine signaling pathway and cytokine-cytokine receptor interaction, implying that the gene family might be important in regulating psoriasis. Luster et al. reviewed that chemokines-chemotactic cytokines could mediate inflammation, which might be the reason for the association between CXC chemokine family and psoriasis [25]. *IVL*, *OASL*, and *ISG15* were also the potential gene targets of the drugs for treating psoriasis in this study. Chen et al. found that *IVL* was related to the early-onset plaque psoriasis [26]. The expression pattern of *OASL* may offer useful information for treating autoimmune diseases and chronic infections [27]. In addition, Raposo et al. observed significant overexpression of 16 antiviral genes in lesional psoriatic skin, with more than two-fold increase in *ISG15* [28].

However, there are some limitations in this study. For instance, the microarray data was obtained from the GEO database, and not generated by the authors. Therefore, further experiments should be conducted to verify whether these target genes can be used in the clinical treatment of psoriasis.

## Conclusions

In the current study, risk subpathway analysis based on DEGs was considered a useful method to identify pathways mostly related to psoriasis. Psoriasis may be mostly caused by keratinization, keratinocyte differentiation, and epidermal cell differentiation and the pathogeneses were more related to pathways such as steroid hormone biosynthesis, folate biosynthesis, and pyrimidine metabolism. Besides, some psoriasis-related genes such as *SPRR* genes, *HSD11B1*, *GGH*, *CXCR2*, *IVL*, *OASL*, *ISG15*, and *CXCL10* may be important targets in psoriatic therapy. Therefore, our study explored the pathogeneses of psoriasis in depth and provided probable target genes for psoriatic therapy.

## Methods

### Microarray data

Gene expression data of human mRNA about psoriasis research (GSE13355 and GSE14905) were obtained from the Gene Expression Omnibus (GEO, http://www.ncbi.nlm.nih.gov/geo/) database. There were 180 samples in GSE13355, including 64 healthy control samples without psoriasis and 116 experimental samples with psoriasis. We chose 64 healthy control samples without psoriasis and 58 skin samples from psoriasis patients in this study. Also, 82 samples from GSE14905, including 21 healthy control samples without psoriasis and 61 experimental samples with psoriasis, among which 21 healthy control samples without psoriasis and 33 skin samples from psoriasis patients were selected in this study. All samples were detected through the Affymetrix Human Genome U133 Plus 2.0 array platform. The sample basic statistical information of GSE13355 and GSE14905 datasets have been shown in Supplementary Table 2 and Supplementary Table 3.

### Data preprocessing and screening of differentially expressed genes

In order to obtain the common gene expression matrix and eliminate the heterogeneity among datasets, we used the ComBat function of sva package [29] of R language to eliminate batch effect between datasets. In addition, the Affy package [30] was applied for preprocessing the obtained data by conducting normalization, background correction and expression calculation. Then the probes were annotated by matrix data combined with chip platform annotation file. The average value of diverse probes would be considered as the eventual expression level of gene if they corresponded to the identical mRNA. Finally, a total of 54,675 probes were mapped to 19,851 gene symbols without repetition.

After data preprocessing, the limma package [31] of R language was utilized to screen DEGs. The false discovery rate (FDR) of Benjamini and Hochberg (BH) method was applied to adjust *p*-values for multiple comparisons. The significant differentially expressed cut-off was set as |logFC| > 2 and adjusted *P* < 0.05. Finally, a heatmap was drawn to observe the clustering of samples.

### Principal component analysis of differentially expressed genes

Principal component analysis (PCA), a multivariate regression analysis, was used to distinguish samples with multiple measurements [32]. A PCA of DEGs was conducted using the ggord package (version: 1.1.4) in R language in the present study. The PCA graph was then obtained, in which DEGs were considered as variables and the differences between normal control and lesional samples were observed.

### GO and KEGG enrichment analysis

The enrichment analysis of DEGs was carried out by utilizing the online software DAVID [33], in which species was set as human and the other parameters were the default values. GO terms with a cut-off of *P* < 0.05 and gene count ≥5 were chosen. Likewise, the KEGG pathways with *P* < 0.05 were selected. Then a plug-in EnrichmentMap in Cytoscape [34] was used to integrate the GO terms with the cutoff of *P <* 0.05 and overlap coefficient larger than 0.5.

### Risk subpathway screening for differentially expressed genes

To obtain more important genes and pathways in psoriasis, the iSubpathwayMiner package (version: 3.0) [35] in R language (version: 3.4.3) was selected to identify the risk subpathways for DEGs. Moreover, the subpathways with *P* < 0.05 were screened, which reflected the pathways involved in psoriasis more clearly.

### Protein-protein interaction network construction

To further analyze the impacts of DEGs on psoriasis, the PPI network was constructed among various DEGs. Also, the online software STRING was used to analyze the interactions of proteins encoded by DEGs [36], and the PPI score was set as 0.4 (referred as median confidence). Then the Cytoscape software [34] (https://cytoscape.org/) was utilized to visualize the PPI network. Finally, the degree of each DEG was obtained by analyzing the topological structure of the PPI network.

### Drug-hub gene interaction

The hub genes with high degree (degree ≥10) of connectivity in PPI were selected as the promising targets for searching drugs through the DGIdb database (version: 3.0.3, http://www.dgidb.org/). This database contains drug-gene interaction data from 30 disparate sources including ChEMBL, DrugBank, Ensembl, NCBI Entrez, PharmGKB, PubChem, clinical trial databases, and literature in NCBI PubMed. The results of this process were arranged such that each entry was a specific drug-gene interaction associated with its source link [37]. The identified target network was visualized using the Cytoscape software.

## Supplementary information

**Additional file 1: Table S1.** Differentially expressed genes between normal and lesional groups.

**Additional file 2: Table S2.** The sample basic statistic information of GSE13355 dataset.

**Additional file 3: Table S3.** The sample basic statistic information of GSE14905 dataset.

## Data Availability

All data generated or analysed during this study are included in this published article.

## Abbreviations

GEO: Gene Expression Omnibus
DEGs: Differentially expressed genes
GO: Gene ontology
KEGG: Kyoto Encyclopedia of Genes and Genomes
PPI: Protein-protein interaction
*HSD11B1*: 11beta-hydroxysteroid dehydrogenase type 1
*GGH*: Gamma-glutamyl hydrolase
